# Comparison of Efficacy of Platelet-Rich Plasma (PRP) With PRP Microneedling in Androgenetic Alopecia

**DOI:** 10.7759/cureus.30418

**Published:** 2022-10-18

**Authors:** Anjum Muhammad, Nadia Iftikhar, Asher Mashhood, Zulqurnain Saleem, Madhia Sundus, Abrar A Khalid, Shumaila Khan, Safoora Naveed, Waseem Shahid, Umair Ajmal, Afnan Akbar

**Affiliations:** 1 Dermatology, Pak Emirates Military Hospital, Rawalpindi, PAK; 2 Medicine, Pak Emirates Military Hospital, Rawalpindi, PAK; 3 Dermatology, President Academy of Advanced Medical Aesthetics, Islamabad, PAK; 4 Radiology, Pakistan Institute of Medical Sciences, Islamabad, PAK; 5 Basic Sciences, Pak Emirates Military Hospital, Rawalpindi, PAK

**Keywords:** platelet-rich plasma (prp), male androgenetic alopecia, prp vs microneedling, hair fall treatment, androgenetic alopecia

## Abstract

Background

Limited data are available on the efficacy of platelet-rich plasma (PRP) and microneedling versus PRP alone. In this study, we aimed to compare the efficacy of PRP and microneedling with PRP alone in androgenetic alopecia (AGA).

Methodology

This prospective, randomized, interventional study was conducted in Pak-Emirates Military Hospital, Rawalpindi, from September 2020 to December 2020. In total, 60 individuals suffering from AGA of up to Hamilton-Norwood grade 4 were randomly assigned to two groups, namely, PRP+microneedling versus PRP alone. A total of three sessions, each one month apart, were offered. Pre and post-intervention photographs, hair count (/cm^2^), hair-pull test, and physician’s and patient’s perception of hair loss were recorded. The data were statistically analyzed.

Results

A greater proportion of patients in the microneedling group achieved a negative hair-pull test and improved perception of hair loss compared to the PRP-alone group (82.1% vs. 51.9% and 88.0% vs. 73.9%, respectively). The percentage increase in mean hair count in the microneedling group (24.53 ± 9.49%) was significantly higher than the increase in the PRP-alone group (17.88 ± 10.15%) (p = 0.011). For grades 2 and 3 hair loss, microneedling+PRP caused a much greater increase in hair count than PRP alone. This difference was less pronounced for Norwood grade 4. No notable side effects were noted in any patient.

Conclusions

Combined PRP and microneedling is more efficacious than PRP alone in patients with AGA up to Hamilton-Norwood grade 4.

## Introduction

Androgenetic alopecia (AGA) is a common disorder affecting more than half of adults worldwide with marked psychosocial impacts on affected individuals [1]. With the underlying pathophysiology still poorly understood, there have been extensive efforts to devise modern and more effective treatment modalities in recent times. Platelet-rich plasma (PRP) therapy is one such well-known intervention for AGA which relies on the growth factors released from platelets. PRP is thought to result in a significant reduction in hair fall in both male and female patients [2]. The treatment is used globally as an adjuvant treatment for AGA alongside topical minoxidil and 5-α reductase inhibitors [3]. There has been a growing need for efforts to determine uniform guidelines for the preparation and administration of PRP and determine optimal combination regimens to enhance its effectiveness in the treatment of AGA [4]. Limited clinical data and experience of the novel modifications to this modality underline the need for more research in this regard. Recently, efforts on international levels have employed a combination of microneedling with PRP for AGA with encouraging results [5]. The concept of microneedling is based on the idea that limited and controlled trauma on the skin would release growth factors and induce the growth of vital dermal elements needed for hair growth and rejuvenation. The study by Kim et al. that utilized mouse models has been reproduced in humans where microneedling was found to have significantly useful effects on hair growth in humans [6]. Our study aims to compare the efficacy of PRP and microneedling combined with conventional PRP alone for AGA in both male and female patients.

## Materials and methods

This randomized, observational, interventional study was conducted at the Department of Dermatology in a tertiary healthcare hospital in Rawalpindi from September 2020 to December 2020 over four months. The study was approved by the ethical committee of the hospital (Ethical committee approval number: A/28/EC/222/2020). The sample size was calculated by the World Health Organization (WHO) calculator, and the non-probability purposive sampling technique was employed for patient selection.

In total, 60 patients were included in the study after obtaining their explicit informed consent. We included patients presenting with AGA up to Hamilton-Norwood grade 4, those fit for PRP/PRP microneedling therapy, and those willing for regular follow-up at the Dermatology Outpatient Department. Patients with alopecia other than AGA; having disorders pertaining to coagulation, hematology, thyroid, and nutrition, as well as dermatological disorders contributing to hair loss; patients unfit for PRP and PRP microneedling therapy; and those who sought hair restoration treatment in the past six months were excluded from the study. The patients were randomly assigned into two groups, namely, the PRP microneedling (group A) and conventional PRP (group B).

PRP preparation was done under aseptic measures, with 20 mL of fresh blood withdrawn via medial cubital venipuncture and mixed with 2 mL of citrate-phosphate-dextrose solution with adenine (CPDA) as an anticoagulant. CPDA included citrate acid anhydrous 0.299 g, sodium citric dehydrate 2.63 g, monobasic sodium phosphate (monohydrate NaH_2_P0_4_H_2_0) 0.222 g, dextrose monohydrate 3.19 g, adenine (C_5_H_5_N_5_) 0.0275, water for injection added to 100 mL, manufactured by Zhejiang Zhongfa Pharmaceuticals Co. Ltd. Zhejiang China. Following the instructions of the American Association of Blood Bank technical manual [7], the blood was centrifuged for 15 minutes with soft spin (1,500 rotations per minute) followed by 10 minutes of hard spin (4,000 rotations per minute). The aim was to obtain a platelet count equal to or more than 10 lakh/mL in 5 mL of PRP, as defined by Marx as a working definition of PRP [8]. Patients unable to obtain this minimum concentration of platelet count were excluded from the study. One part of calcium gluconate was added to nine parts of PRP, as a platelet activator, before injection/topical administration.

In Group A, microneedling was done using a derma roller with needles measuring 2 mm on all plains of the scalp till erythema/pinpoint bleeding was observed as an endpoint. Following microneedling, PRP was applied topically and massaged thoroughly to the target area in group A. In group B, only PRP was injected via 1 mL insulin syringes using the nappage technique in the target area following proper aseptic measures.

The AGA grade was recorded using the Hamilton-Norwood scoring system [9]. Pre and post-treatment assessment was done in both groups via photograph (using standard light settings and angles using a DSLR camera), hair-pull test, and hair count in a predefined fixed 1 cm^2^ area of the scalp, which was determined by drawing a straight line from the medial canthus onto the scalp and the upper border of the tragus. The point at which both lines crossed was taken, as the center of a 1 cm^2^ box, which was noted for each patient. Hair follicles were counted in this area three times, and an average of three counts was calculated and noted at the start and end of the study. At the end of the treatment, photographs, hair count/cm^2^ area, hair-pull results, and patients’ level of satisfaction were noted for both groups. A specially designed proforma was employed to assess and compare patients’ level of satisfaction and pain severity perception. Patient satisfaction was measured on a scale of 1-10 (using a Likert scale, further stratification was done, where 1‐3 = no satisfaction, 4‐6 = moderate satisfaction, and greater than 7 = high satisfaction).

Data analysis was done using SPSS version 23 (IBM Corp., Armonk, NY, USA). P-values were calculated for relevant variables.

## Results

Of the total of 60 patients included in the study, 57 were male and three were female. The mean age of the participants was 29.2 ± 6.43 years. The descriptive characteristics of the study sample are presented in Table 1, which also depicts the pain severity scoring categories reported by each of the study groups. Patients in the PRP-alone group were significantly more likely to report higher pain perception than those in the microneedling group (p < 0.001). No other notable side effects were noted in any patient.

**Table 1:** Descriptive characteristics of the study population. PRP: platelet-rich plasma

Descriptive characteristics		Treatment received		Total
		PRP alone	Microneedling + PRP	
Age (years)		28.17 ± 5.41	30.23 ± 7.25	29.2 ± 6.43
Gender	Male	27 (47.4%)	30 (52.6%)	57 (100.0%)
	Female	3 (100.0%)	0 (0.0%)	3 (100.0%)
Hamilton-Norwood grade of alopecia on induction	Grade 1	0 (0.0%)	0 (0.0%)	0 (0.0%)
	Grade 2	13 (59.1%)	9 (40.9%)	22 (100.0%)
	Grade 3	15 (46.9%)	17 (53.1%)	32 (100.0%)
	Grade 4	2 (33.3%)	4 (66.7%)	6 (100.0%)
Pain severity perception	Mild	4 (16.0%)	21 (84.0%)	25 (100.0%)
	Moderate	14 (60.9%)	9 (39.1%)	23 (100.0%)
	Severe	10 (100.0%)	0 (0.0%)	10 (100.0%)
	Very Severe	2 (100.0%)	0 (0.0%)	2 (100.0%)
Total		30 (50.0%)	30 (50.0%)	60 (100.0%)

Clinical and photographic comparison of the two groups at the end of the study period revealed better treatment results in group A compared to group B.

McNemar analyses were done to compare the outcomes of each intervention after three months compared to baseline values. Two parameters were used for this assessment, namely, the hair-pull test and self-reported perception of hair loss, which are represented in Table 2. Overall, a significant change from positive to negative hair-pull test was seen in both treatment groups (p < 0.001). However, a greater proportion of 82.1% of patients in group A (microneedling group) managed to achieve these results in contrast to 51.9% in group B (PRP alone). In both groups, there were no patients who showed positive hair-pull tests after initially testing negative at baseline.

**Table 2:** McNemar contingency table for the self-reported perception of hair loss at baseline and at the end of the intervention in relation to treatment received. PRP: platelet-rich plasma

Treatment received			Hair loss perception at the end of treatment		Total
			Mild/Moderate	Severe	
PRP alone	Hair loss perception at baseline	Mild/Moderate	7 (100.0%)	0 (0.0%)	7 (100.0%)
		Severe	17 (73.9%)	6 (26.1%)	23 (100.0%)
	Total		24 (80.0%)	6 (20.0%)	30 (100.0%)
Microneedling + PRP	Hair loss perception at baseline	Mild/Moderate	5 (100.0%)	0 (0.0%)	5 (100.0%)
		Severe	22 (88.0%)	3 (12.0%)	25 (100.0%)
	Total		27 (90.0%)	3 (10.0%)	30 (100.0%)
Total	Hair loss perception at baseline	Mild/Moderate	12 (100.0%)	0 (0.0%)	12 (100.0%)
		Severe	39 (81.3%)	9 (18.8%)	48 (100.0%)
	Total		51 (85.0%)	9(15.0%)	60 (100.0%)

**Figure 1:**
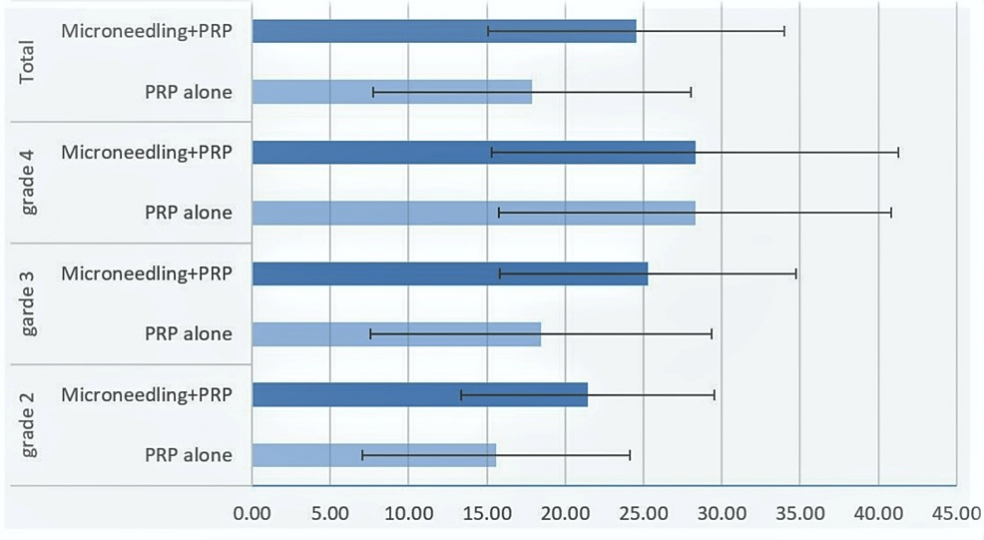
Mean percentage improvement in hair count in relation to treatment received and Norwood grade. PRP: platelet-rich plasma

The percentage increase in mean hair count after treatment was calculated for patients in both treatment groups. Overall, patients in the microneedling group had an increase of 24.53 ± 9.49%, which was significantly higher than the 17.88 ± 10.15% increase seen in the PRP-alone group (p = 0.011). Figure 1 represents these differences graphically. Figure 1 also shows the percentage increases in mean hair counts for patients within each individual Norwood grade. For grades 2 and 3 hair loss, microneedling and PRP are shown to cause a much greater increase in hair count than PRP alone. This difference is less pronounced for Norwood grade 4.

## Discussion

PRP has become a widely employed treatment modality for hair restoration and rejuvenation in recent years. The evidence for its effectiveness, although variable, remains largely convincing [4].

While the need for uniform, well-structured guidelines for the preparation of PRP remains crucial, the efforts to increase the efficacy of this procedure in AGA deserve equal attention.

The mechanism of hair rejuvenation in PRP has been postulated by various studies. Apart from the in vitro studies, which reported enhanced dermal papilla cell growth via activating extracellular kinase signaling, the release of various growth factors from the activated platelets may also result in an enhanced β‐catenin transcriptional activity and a robust differentiation of stem cells into hair follicle cells [5,10]. Various studies have reported an increase in the ratio of anagen hair compared to catagen hair in the affected scalp [4]. Microneedling is thought to induce micro-trauma in a controlled environment to the skin and exert a similar rejuvenation effect via the release of various growth factors, thereby inducing hair growth and preventing hair fall [10]. Faster hair growth with a shinier texture was reported by Kim et al. in the microneedling-treated mice group compared to controls [6].

Our study aims to corroborate the findings of previous studies which reported an enhanced response to PRP combined with microneedling. Studies by Jha et al. and Dhurat et al. reported similar results [5,10]. The amount of work to ascertain the combined efficacy of microneedling with PRP on the Asian front, in general, and in Pakistan, in particular, remains scant to say the least. Our study strives to fill this gap by reporting a Pakistani experience with this combination treatment modality for AGA.

Combining PRP with microneedling results in better hair count, a higher frequency of negative hair-pull tests, and improved results as per physician and patient-reported assessment. Pain perception is also lower in combination therapy compared to conventional PRP. Lesser pain may ensure better compliance and, hence, better treatment outcomes. However, there are a few drawbacks of the combined approach, namely, the higher amount of procedure time required for microneedling and the relatively arduous nature of performing microneedling in patients with long hair. The procedure may especially be cumbersome in patients (especially females) with long hair where hair could get stuck in the derma roller/microneedling device. In males, this difficulty could be surmounted by keeping the hair short during the treatment period or just before the procedure; however, this may still be unacceptable to many male patients.

Study limitations

The limitations of our study include a smaller sample size, a relative underrepresentation of female patients, and the lack of dermoscopy/histopathology to assess hair growth pre and post-treatment.

## Conclusions

Our study concludes that a combined PRP and microneedling treatment may be more effective than PRP alone in the treatment of AGA. The combined approach may also ensure better patient satisfaction and compliance compared to the conventional approach, especially because the microneedling technique is less painful. Decreased pain severity scores associated with the microneedling arm could improve patient compliance and translate into better treatment success rates. However, microneedling PRP may require more time that its conventional counterpart. Microneedling could also marginally increase the cost of the procedure for patients.

The efficacy of both modalities may increase if the intervention is offered at an earlier stage of hair loss. This is the first study of its kind in Pakistan and may be followed by further large-scale studies to substantiate our findings.

## References

[1] Mian M, Silfvast-Kaiser A, Paek S, Kivelevitch D, Menter A (2019). A review of the most common dermatologic conditions and their debilitating psychosocial impacts. Int Arch Int Med.

[2] York K, Meah N, Bhoyrul B, Sinclair R (2020). A review of the treatment of male pattern hair loss. Expert Opin Pharmacother.

[3] Gupta AK, Venkataraman M, Talukder M, Bamimore MA (2022). Relative efficacy of minoxidil and the 5-α reductase inhibitors in androgenetic alopecia treatment of male patients: a network meta-analysis. JAMA Dermatol.

[4] Badran KW, Sand JP (2018). Platelet-rich plasma for hair loss: review of methods and results. Facial Plast Surg Clin North Am.

[5] Jha AK, Udayan UK, Roy PK, Amar AK, Chaudhary RK (2018). Original article: platelet-rich plasma with microneedling in androgenetic alopecia along with dermoscopic pre- and post-treatment evaluation. J Cosmet Dermatol.

[6] Kim BJ, Lim YY, Kim HM, Lee YW, Won CH, Huh CH, Kang H (2012). Hair follicle regeneration in mice after wounding by microneedle roller. Int J Trichol.

[7] American Association of Blood Banks Technical Manual Committee (1999). Method 6.11: preparation of platelets from whole blood. AABB Technical Manual.

[8] Marx RE (2001). Platelet-rich plasma (PRP): what is PRP and what is not PRP?. Implant Dent.

[9] Gupta M, Mysore V (2016). Classifications of patterned hair loss: a review. J Cutan Aesthet Surg.

[10] Dhurat R, Sukesh M, Avhad G, Dandale A, Pal A, Pund P (2013). A randomized evaluator blinded study of effect of microneedling in androgenetic alopecia: a pilot study. Int J Trichology.

